# Optimal partner wavelength combination method applied to NIR spectroscopic analysis of human serum globulin

**DOI:** 10.1186/s13065-020-00689-z

**Published:** 2020-05-24

**Authors:** Yun Han, Yun Zhong, Huihui Zhou, Xuesong Kuang

**Affiliations:** 1 Department of Data Science, Guangdong Ocean University, Haida Road 1, Mazhang District, Zhanjiang, 524088 China; 2 Zhanjiang No. 2 High School Hai Dong, Potou District, Zhanjiang, 524057 China

**Keywords:** Optimal partner wavelength combination, Near-infrared spectroscopy, Human serum globulin

## Abstract

Human serum globulin (GLB), which contains various antibodies in healthy human serum, is of great significance for clinical trials and disease diagnosis. In this study, the GLB in human serum was rapidly analyzed by near infrared (NIR) spectroscopy without chemical reagents. Optimal partner wavelength combination (OPWC) method was employed for selecting discrete information wavelength. For the OPWC, the redundant wavelengths were removed by repeated projection iteration based on binary linear regression, and the result converged to stable number of wavelengths. By the way, the convergence of algorithm was proved theoretically. Moving window partial least squares (MW-PLS) and Monte Carlo uninformative variable elimination PLS (MC-UVE-PLS) methods, which are two well-performed wavelength selection methods, were also performed for comparison. The optimal models were obtained by the three methods, and the corresponding root-mean-square error of cross validation and correlation coefficient of prediction (SECV, R_P,CV_) were 0.813 g L^−1^ and 0.978 with OPWC combined with PLS (OPWC-PLS), and 0.804 g L^−1^ and 0.979 with MW-PLS, and 1.153 g L^−1^ and 0.948 with MC-UVE-PLS, respectively. The OPWC-PLS and MW-PLS methods achieved almost the same good results. However, the OPWC only contained 28 wavelengths, so it had obvious lower model complexity. Thus it can be seen that the OPWC-PLS has great prediction performance for GLB and its algorithm is convergent and rapid. The results provide important technical support for the rapid detection of serum.

## Introduction

Near infrared (NIR) spectroscopy is a green and developing analytical technique, which has been widely used in life sciences [1–7], agricultural products and food [8–11], soil [12–14], and other fields [15, 16]. For NIR spectroscopic analysis of complex system, wavelength selection is necessary and difficult. So far, many methods including continuous mode and discrete mode of wavelength selection have been successfully used in NIR spectroscopy analysis, but a general and effective method has not been found. Moving window partial least squares (MW-PLS) is a widely used and well performed wavelength selection method, which uses a moving window whose position and size can be changed to identify and select continuous wavebands in terms of the prediction effect, and such waveband can correspond to absorption of specific functional groups [13, 15, 16]. This method can achieve high prediction effect on most spectral data sets, so it often presents as the comparison method of new method to evaluate the performance of the new method. However, it can be seen from the papers [16–18], as a traversal algorithm for continuous wavebands, all possible continuous bands are screened, this method is time-consuming when encountering a large dataset. Monte Carlo uninformative variable elimination by PLS (MC-UVE-PLS) is a popular method for discrete wavelength selection [19], which creatively introduced noise to eliminate uninformative variables, but it cannot achieve satisfactory prediction results for some data sets.

Serum globulin (GLB), which is synthesized by human monocyte-phagocyte system, contains various antibodies in the serum of healthy people, so it can enhance the body’s resistance to prevent infection. It is mainly used for immunodeficiency diseases as well as prevention and treatment of viral infections and bacterial infections such as infectious hepatitis, measles, chickenpox, mumps and herpes zoster. In addition, it can also be used in asthma, allergic rhinitis, eczema and other endogenous allergic diseases. Therefore, the GLB in human serum is very important for clinical trials and disease diagnosis. In previous studies [20, 21], FTIR/ATR spectroscopy was used for determination of GLB. The study found that for blood index, the NIR has higher quantitative analysis accuracy than the FTIR/ATR spectroscopy [6, 22]. The experimental results show that the molecular absorption information of GLB can be captured by NIR spectroscopy without reagent.

Optimal partner wavelength combination (OPWC) is a method of selecting discrete information wavelength by iteration. For the method, the best partner of each wavelength in a predetermined wavelength region was determined based on binary linear regression (BLR), and a partner wavelength subset (PWS) was obtained; then the best partner of each wavelength in the PWS was obtained with the same method. The iterative process may be continued until convergence was met, and the last obtained wavelength subset was called OPWC. On the basis of the OPWC, PLS model was established. In order to make full use of the samples, the leave-one-out cross validation (LOOCV) was adopted.

Because human serum is a complex multi-component system and the absorption interference of other components is very complex, it is difficult to extract the characteristic information of GLB. Therefore, OPWC-PLS method was employed to remove redundant wavelength and establish a high precision quantitative model. MW-PLS and MC-UVE-PLS methods were also performed for comparison. Experimental results showed that the OPWC-PLS has great prediction performance and the algorithm is convergent and rapid.

## Materials and methods

### Experiment

A total of 230 human serum samples were collected in this experiment and their GLB values were determined using routine clinical biochemical tests. This work was supported by Youth Innovation Talents Project of Colleges and Universities in Guangdong Province (No. Q18285), and all individual participants provided written informed consent. The study protocol was performed in accordance with relevant laws and institutional guidelines and was approved by local medical institutions and ethics committee. The obtained results were used as reference values in NIR spectroscopy analysis. The statistical analysis of the measured GLB values of 230 samples is given in Table 1.

**Table 1:** Statistical analysis of measured GLB values of 230 samples

Indicator	Number	Min	Max	Mean	SD
GLB(g L^−1^)	230	18.70	41.60	27.477	3.953

The spectroscopy instrument was an XDS Rapid Content™ Liquid Grating Spectrometer (FOSS, Denmark) equipped with a transmission accessory and a 2 mm cuvette. The spectral scanning range was 780-2498 nm with a 2 nm wavelength gap; the detector were Si (780–1100 nm) and Pbs (1100–2498 nm). The temperature and relative humidity of the laboratory were 25 ± 1 °C and 46 ± 1% RH, respectively. Each sample was measured three times, and the mean value of the three measurements was used for modeling.

### Modeling process

Leave-one-out cross validation (LOOCV) is commonly used as the object function for model selection, which aims to make full use of the samples information. In this study, LOOCV was conducted for modeling process, as described below. Only one sample was left out from modeling samples for the prediction, and the other samples were used as calibration set. This process was repeated until the prediction value of every modeling sample was obtained. The measured and predicted values of *i*th sample in modeling set were denoted as $$ C_{{{\text{M}},{\kern 1pt} {\kern 1pt} i}} , $$$$ \tilde{C}_{{{\text{M}},{\kern 1pt} {\kern 1pt} i}} , $$$$ i = 1,{\kern 1pt} {\kern 1pt} \;2, \ldots ,\;n_{\text{M}} , $$$$ n_{\text{M}} $$ was the number of modeling samples. For all samples, the mean measured value was denoted as $$ C_{{{\text{M,}}{\kern 1pt} {\kern 1pt} {\kern 1pt} {\text{Ave}}}}^{{}} , $$ and the mean predicted value was denoted as $$ \tilde{C}_{{{\text{M}},{\kern 1pt} {\kern 1pt} {\text{Ave}}}}^{{}} $$. The prediction accuracy was evaluated by the root-mean-square errors of cross validation and the predicted correlation coefficients, and denoted as SECV and R_P,CV_, respectively. The calculation formulas were as the follows:

1 $$ {\text{SECV }} = \sqrt {\frac{{\sum\nolimits_{i = 1}^{{n_{M} }} {(\tilde{C}_{{{\text{M,}}{\kern 1pt} {\kern 1pt} {\kern 1pt} i}}^{{}} - C_{{{\text{M,}}{\kern 1pt} {\kern 1pt} {\kern 1pt} {\kern 1pt} i}}^{{}} )^{2} } }}{{n_{M} }}} , $$

2 $$ {\text{R}}_{\text{P, CV}} = \frac{{\sum\nolimits_{i = 1}^{{n_{M} }} {(C_{{{\text{M,}}{\kern 1pt} {\kern 1pt} {\kern 1pt} i}}^{{}} - C_{{{\text{M,}}{\kern 1pt} {\kern 1pt} {\kern 1pt} {\text{Ave}}}}^{{}} )(\tilde{C}_{{{\text{M,}}{\kern 1pt} {\kern 1pt} {\kern 1pt} i}}^{{}} - \tilde{C}_{{{\text{M,}}{\kern 1pt} {\kern 1pt} {\kern 1pt} {\text{Ave}}}}^{{}} )} }}{{\sqrt {\sum\nolimits_{i = 1}^{{n_{M} }} {(C_{{{\text{M,}}{\kern 1pt} {\kern 1pt} {\kern 1pt} i}}^{{}} - C_{{{\text{M,}}{\kern 1pt} {\kern 1pt} {\kern 1pt} {\text{Ave}}}}^{{}} )^{2} (\tilde{C}_{{{\text{M,}}{\kern 1pt} {\kern 1pt} {\kern 1pt} i}}^{{}} - \tilde{C}_{{{\text{M,}}{\kern 1pt} {\kern 1pt} {\kern 1pt} {\text{Ave}}}}^{{}} )^{2} } } }} $$

The model parameters were selected to achieve minimum SECV.

### MW-PLS method

MW-PLS is a time-tested and popular method for screening continuous wavebands. This method uses several continuous wavelengths as a window, the size and position of which can be changed, and the PLS models are established for all possible windows in a predetermined search region of the spectrum. The information waveband was selected according to the minimum SECV. In this study, the search range of the MW-PLS was full spectrum region (780–2498 nm) with 860 wavelengths, and the initial wavelength (*I*) and number of wavelengths (*N*) of window as well as the number of PLS factors (*F*) were set as $I \in \{ 780,\;782, \ldots ,\;2498\}$, $N \in \{ 1,\;2, \ldots ,\;200\} \cup \{ 210,\;220, \ldots ,\;860\}$, and $F \in \{ 1,\;2, \ldots ,20\}$. The LOOCV for PLS models was performed in each combination of (*I*, *N*, *F*), and the corresponding SECV and R_P,CV_ were calculated. The optimal waveband with minimum SECV was selected to achieve the best prediction accuracy.

### MC-UVE-PLS method

MC-UVE-PLS is a representative method for screening discrete wavelengths. For the method, lots of models are established with randomly selected calibration samples, then the coefficient stability of these models is calculated, and each variable is evaluated with the stability of the corresponding coefficient [19]. In this study, MC-UVE method was performed based on the full spectrum region, and Monte Carlo sampling operation 500 times. The number of variables was determined using the method in Ref. [19]. MC-UVE-PLS was rerun for 50 times and the best result was recorded for further analysis. The number of PLS factors *F* was set to be $F \in \{ 1,\;2, \ldots ,30\}$.

### OPWC-PLS method

Based on BLR, the best partner of each wavelength was screened for entire scanning region and a partner wavelength subset (PWS) is determined. Then, a new PWS of all wavelengths in the PWS are also determined according to above obtained correspondence. The same procedure was performed repeatedly until the results converged to optimal partner wavelength combination (OPWC). The specific steps are as follows:

*Step 1* Assume that there are *N* wavelengths in the wavelength screening area $\Delta$, namely, $\Delta = \left\{ {\lambda_{1} ,\,\lambda_{2} , \ldots ,\,{\kern 1pt} \lambda_{N} {\kern 1pt} } \right\}$. For any fixed $\lambda_{i} \in \Delta$, and $\forall \lambda_{k} \in \Delta ,{\kern 1pt} {\kern 1pt} \;{\kern 1pt} k \ne i$, LOOCV was performed based on binary linear regression of wavelength combination $(\lambda_{i} ,{\kern 1pt} \,\lambda_{k} )$. The best partner of $\lambda_{i}$ was identified and denoted as $f(\lambda_{i} )$ based on minimum ${\text{SECV}}(\lambda_{i} ,{\kern 1pt} \lambda_{k} )$. The formula is as follows,

$$ {\text{SECV}}(\lambda_{i} ,{\kern 1pt} f(\lambda_{i} )) = \mathop {\hbox{min} }\limits_{\begin{subarray}{l} k = 1,2, \cdots ,N \\ k \ne i \end{subarray} } {\text{SECV}}(\lambda_{i} ,{\kern 1pt} \lambda_{k} ) $$

The $f(\Delta )$ was partner wavelength subset (PWS^(1)^) of $\Delta$, and its number of wavelengths was denoted by *N*^(1)^. Theoretically, the best partner $f(\lambda_{i} )$ for each wavelength $\lambda_{i}$ is unique, but several different wavelengths may have the same best partner. If some $\lambda$ was not a best partner of any wavelength, then $\lambda \notin$ PWS^(1)^, and *N*^(1)^ < *N*.

*Step* 2 According to the projection $f$ defined above, the partner wavelength subset (PWS^(2)^) of PWS^(1)^ could be obtained. It will be proved later that PWS converges to stable number of wavelengths after finite projection iterations. Suppose that PWS converges after *s*-times iterations, *N*^(*s*)^ = *N*^(*s*+1)^. And the PWS^(s)^ was called optimal partner wavelength combination (OPWC). For OPWC, each wavelength was the best partner of some other wavelength.

### The proof of convergence of algorithm

#### *Proof*

(1) If $\forall {\kern 1pt} {\kern 1pt} i,{\kern 1pt} {\kern 1pt} j,{\kern 1pt} {\kern 1pt} {\kern 1pt} {\kern 1pt} i \ne j,{\kern 1pt} {\kern 1pt} {\kern 1pt} \lambda_{i} \ne \lambda_{j}$, $f(\lambda_{i} ) \ne {\kern 1pt} f(\lambda_{j} )$, then the projection $f$ is a one-to-one mapping function defined on $\Delta$, $f(\Delta ) = \Delta$, i.e. the PWS stop shrinking after this projection.

(2) If $$ \exists {\kern 1pt} {\kern 1pt} {\kern 1pt} i,{\kern 1pt} {\kern 1pt} j,{\kern 1pt} {\kern 1pt} {\kern 1pt} {\kern 1pt} i \ne j,{\kern 1pt} {\kern 1pt} \lambda_{i} \ne \lambda_{j} $$, $$ f(\lambda_{i} ) = f(\lambda_{j} ) $$, then $$ f(\Delta ) $$ is a proper subset of $$ \Delta $$, which is set as $$ f(\Delta )\; = \;\left\{ {f(\lambda_{i} )\left| {\lambda_{i} \in \Delta } \right.\} = \{ \lambda_{ 1}^{ ( 1 )} ,\lambda_{ 1}^{ ( 1 )} , \ldots \lambda_{{N^{(1)} }}^{ ( 1 )} } \right\} $$, *N*^(1)^ < *N*. Next further consider the projection of $$ f(\Delta ) $$, i.e.$$ f^{(2)} (\Delta ) $$: (a) If $$ \forall {\kern 1pt} {\kern 1pt} i,{\kern 1pt} {\kern 1pt} j,{\kern 1pt} {\kern 1pt} {\kern 1pt} {\kern 1pt} i \ne j,{\kern 1pt} {\kern 1pt} \lambda_{i}^{(1)} \ne \lambda_{j}^{(1)} $$, $$ f(\lambda_{i}^{(1)} ) \ne {\kern 1pt} f(\lambda_{j}^{(1)} ) $$, then function $$ f $$ is a one-to-one mapping defined on the $$ f(\Delta ) $$, $$ f^{(2)} (\Delta ) = f(\Delta ) $$, i.e. the PWS stop shrinking after this projection. b) If $$ \exists {\kern 1pt} {\kern 1pt} {\kern 1pt} {\kern 1pt} i,{\kern 1pt} {\kern 1pt} j,{\kern 1pt} {\kern 1pt} {\kern 1pt} {\kern 1pt} i \ne j,{\kern 1pt} {\kern 1pt} {\kern 1pt} \lambda_{i}^{(1)} \ne \lambda_{j}^{(1)} ,{\kern 1pt} {\kern 1pt} {\kern 1pt} f(\lambda_{i}^{(1)} ) = f(\lambda_{j}^{(1)} ),{\kern 1pt} $$ then $$ f^{(2)} (\Delta ) $$ is a proper subset of $$ f(\Delta ) $$, which is set as $$ f^{(2)} (\Delta ) = \left\{ {f(\lambda_{i}^{(1)} )\left| {\lambda_{i}^{(1)} \in f(\Delta )} \right.} \right\} $$$$ = \left\{ {\lambda_{ 1}^{ ( 2 )} ,{\kern 1pt} {\kern 1pt} \,\lambda_{ 2}^{ ( 2 )} ,{\kern 1pt} \ldots ,{\kern 1pt} \,\lambda_{{N^{(2)} }}^{ ( 2 )} } \right\} $$, *N*^(2)^ < *N*^(1)^ < *N*.

Similarly considered the projection of $$ f^{(s - 1)} (\Delta ) $$, i.e.$$ f^{(s)} (\Delta ) $$: (a) If $$ \forall {\kern 1pt} {\kern 1pt} i,{\kern 1pt} {\kern 1pt} j,{\kern 1pt} {\kern 1pt} {\kern 1pt} {\kern 1pt} i \ne j,{\kern 1pt} {\kern 1pt} \lambda_{i}^{(s - 1)} \ne \lambda_{j}^{(s - 1)} $$, $$ f(\lambda_{i}^{(s - 1)} ) \ne {\kern 1pt} f(\lambda_{j}^{(s - 1)} ) $$, then the function $$ f $$ is a one-to-one mapping defined on the $$ f^{(s - 1)} (\Delta ) $$, $$ f^{(s)} (\Delta ) = f^{(s - 1)} (\Delta ) $$, i.e. the PWS stop shrinking after this projection. (b) If $$ \exists {\kern 1pt} {\kern 1pt} {\kern 1pt} i,{\kern 1pt} {\kern 1pt} j,{\kern 1pt} {\kern 1pt} {\kern 1pt} {\kern 1pt} i \ne j,{\kern 1pt} {\kern 1pt} \lambda_{i}^{(s - 1)} \ne \lambda_{j}^{(s - 1)} $$, $$ f(\lambda_{i}^{(s - 1)} ) = f(\lambda_{j}^{(s - 1)} ),{\kern 1pt} $$ then $$ f^{(s)} (\Delta ) $$ is a proper subset of $$ f^{(s - 1)} (\Delta ) $$, which is set as $$ f^{(s)} (\Delta ) = \{{f(\lambda_{i}^{(s - 1)} )}\left| {\lambda_{i}^{(s - 1)} \in f^{(s - 1)} (\Delta )} \right.\} $$$$ = \{ \lambda_{ 1}^{({\text{s)}}}, \lambda_{ 2}^{{ ( {\text{s)}}}}, \ldots, \lambda_{{N^{(s)} }}^{{ ( {\text{s)}}}} \}  $$,$$ N^{(s)} < N^{(s - 1)} < \cdots < N $$. Because the total number of wavelengths (*N*) is limited, the number of projections needed is limited.

In this study, the wavelength screening region for GLB spanned the entire scanning region (780–2498 nm), i.e. $\Delta = \left\{ {780,{\kern 1pt} {\kern 1pt} 782, \ldots ,{\kern 1pt} {\kern 1pt} 2498} \right\}$, with 860 wavelengths. The number of PLS factors *F* was set to $F \in \{ 1,\,2, \ldots ,\,20\}$.

The computer algorithms for the three methods discussed above were designed using MATLAB version 7.6.

## Results and discussion

### Results with MW-PLS

**Fig. 1:**
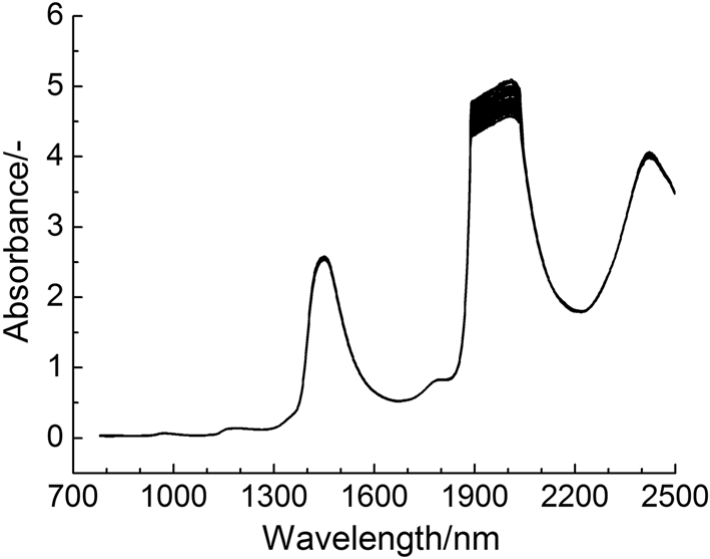
NIR spectra of 230 human serum samples in the scanning area (780–2498 nm)

The NIR spectra of 230 human serum samples in the scanning area (780–2498 nm) were shown in Fig. 1. As can be seen from the figure, absorption at about 2000 nm and 2400 nm has obviously strong noise. In order to obtain satisfactory results, wavelength selection must be carried out to overcome noise interference. For comparison, PLS model of the full spectrum region was first established. The corresponding SECV and R_P,CV_ were 1.423 g L^−1^ and 0.935, respectively.

MW-PLS method was performed to optimize waveband and improve prediction accuracy. Depending on minimum SECV value, the optimal MW-PLS model was selected out. The corresponding waveband was 1504 to 1820 nm, located in the long-NIR region (1100 to 2498 nm). Prediction effects (SECV and R_P,CV_) and parameters of the above two methods were summarized in Table 2. The results showed that the predicted values were highly correlated with clinical measurements for the two methods, and comparing with optimal PLS model in full spectrum region, the optimal MW-PLS model achieved better prediction effect with fewer wavelengths.

**Table 2:** Prediction effects of three methods

Methods	Adopted wavelengths (nm)	*N*	*F*	SECV	R_P,CV_
PLS	780–2498	860	15	1.423	0.935
MW-PLS	1504–1820	159	10	0.804	0.979
OPWC-PLS	1410, 1534, 1536, 1538, 1542, 1676, 1678, 1698, 1732, 1734, 1738, 1742, 1744, 1746, 1750, 1870, 2128, 2132, 2218, 2220, 2222, 2228, 2254, 2258, 2306, 2310, 2318, 2340	28	7	0.813	0.978

### Results with MC-UVE-PLS

The MC-UVE method was performed for eliminating the uninformative variables. Based on the parameter settings in section “MC-UVE-PLS method”, 180 wavelengths were selected, and the SECV and R_P,CV_ for the corresponding PLS models were 1.153 g L^−1^ and 0.948, respectively. Compared with the result of PLS in the full spectrum range, the prediction ability of this method was not significantly improved, which may be because it only eliminates non information variables without considering the influence of interference variables, while serum is a complex system with multiple interference variables.

### Results with OPWC-PLS

**Fig. 2:**
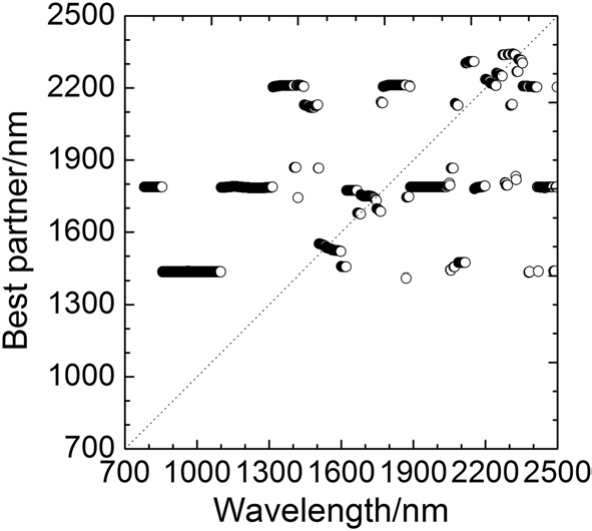
Best partners of 860 wavelengths in the full spectrum region

The OPWC method was performed for screening information wavelength based on the steps mentioned in section “OPWC-PLS method”. Firstly, 104 best partners for all 860 wavelengths were determined according to the results of LOOCV-BLR analysis, and PWS^(1)^ with 104 wavelengths was obtained. Thus, the number of wavelengths was greatly reduced after the first projection. The correspondence between all 860 wavelengths and their best partners was shown in Fig. 2. As shown in the figure, some wavelengths had the same best partner, such as the 2156 nm and 2190 nm as best partners of other wavelengths appeared 3 and 8 times, respectively, so projection $f$ was not a one-to-one mapping function in the whole spectral region $\Delta$. Obviously, $f(\Delta )$ was a subset of $\Delta$ and the projection continues.

**Fig. 3:**
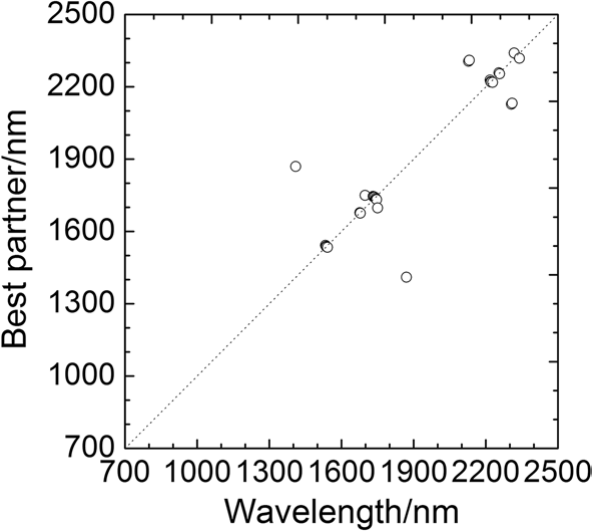
Best partners of the selected 28 wavelengths

Based on the corresponding relationship determined above, the best partner of $\lambda_{i}^{(1)}$ was easy to be selected, and the PWS^(2)^ was obtained. Repeated the same process for PWS^(2)^, and PWS^(3)^ was obtained. As the projection progresses, the number of wavelengths decreased gradually until the number of wavelengths for PWS^(6)^ no longer changed. The PWS^(6)^ was the OPWC and it had only 28 wavelengths. Figure 3 showed the 28 wavelengths and their best partners. As the figure showed, the 28 wavelengths are divided into 14 groups, and the two wavelengths in each group are the best partners for each other.

**Fig. 4:**
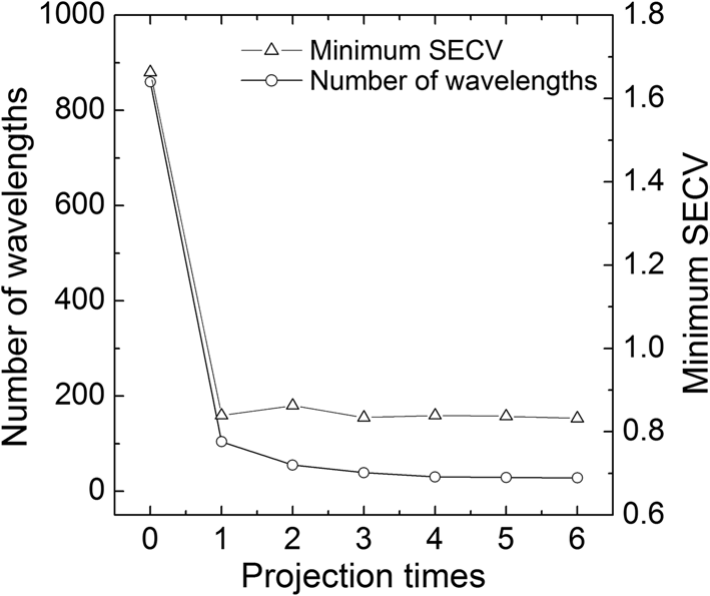
Number of wavelengths and minimum SECV value for each projection

Based on PLS, the LOOCVs were performed for every PWS, and the corresponding minimum SECV value and number of wavelengths (*N*^(*s*)^) used are shown in Fig. 4. As shown in the figure, the *N*^(*s*)^ and minimum SECV values have almost the same trend. After the first projection, both of them decrease rapidly, and the remaining wavelengths are more important, so as the number of projections increases, they slowly decrease. This may be due to the removal of a large amount of noise and background information from the original spectrum after the first projection, so both the *N*^(*s*)^ and minimum SECV values decrease rapidly. The partner wavelength subset of the original spectrum contains less redundant information, so the *N*^(*s*)^ and minimum SECV values decrease slowly in the later projection iteration.

### Comparison of OPWC-PLS and MW-PLS methods

**Fig. 5:**
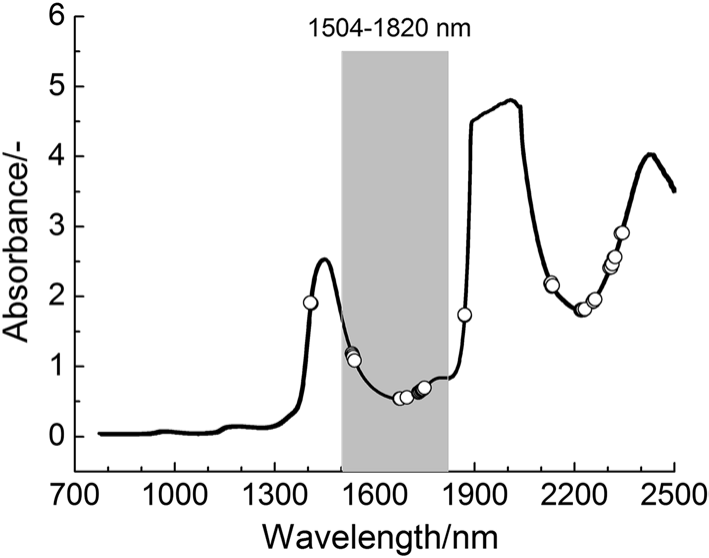
Position of the selected wavelengths with MW-PLS and OPWC-PLS located the average spectrum

Screening the information wavelengths of GLB in the human serum of a multi-component complex system is difficult and complicated. The wavelengths selected by the OPWC-PLS and MW-PLS methods, which correspond to the information of GLB, were shown in Fig. 5. As indicated in Fig. 5, the wavelengths selected by the OPWC method have a wider distribution range and partially coincides with the wavelengths selected by MW-PLS. This may be because the local characteristics of MW-PLS method make some wavelengths cannot be detected, which reflects the complexity of NIR model optimization and the commonness and difference of different methods.

**Fig. 6:**
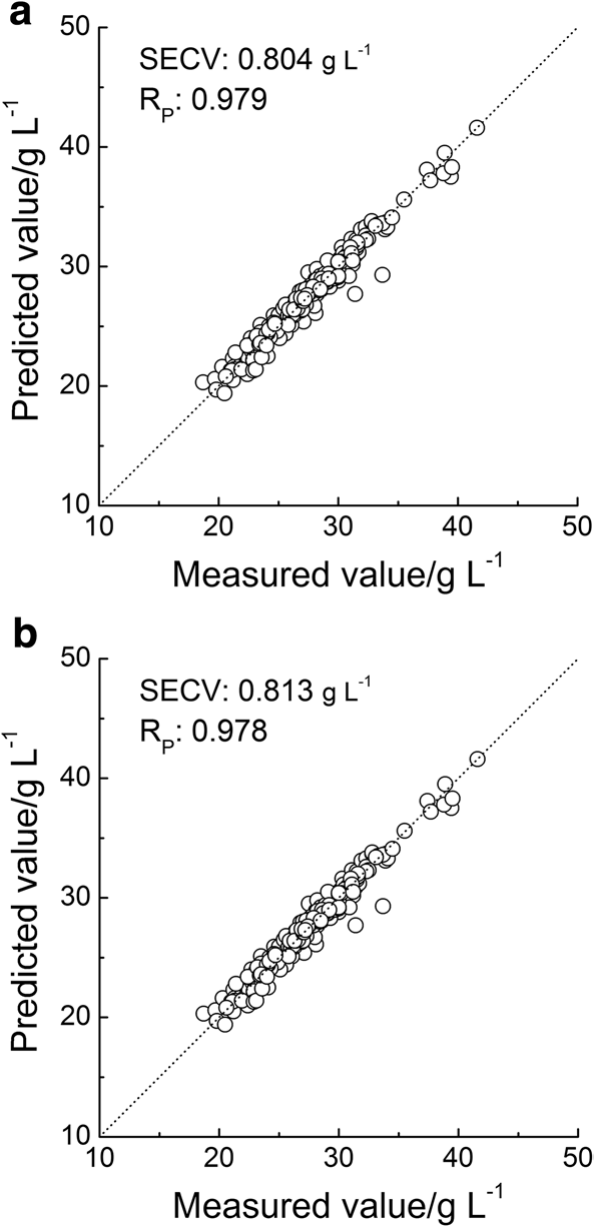
Relationship between the predicted values and measured values of GLB based on **a** MW-PLS and **b** OPWC-PLS methods

Figure 6 showed the relationship between the predicted and measured GLB values based on the MW-PLS and OPWC-PLS methods, respectively. The prediction effect and corresponding parameters *N* and *F* were summarized in Table 2. The SECV and R_P,CV_ were 0.813 g L^−1^ and 0.978 with OPWC-PLS, and 0.804 g L^−1^ and 0.979 with MW-PLS, respectively. The results show that, like MW-PLS, the prediction effect of OPWC-PLS was also obviously better than that of the whole spectrum PLS, and the OPWC is an effective method for screening wavelengths. The phenomenon conveys that better prediction results can be achieved with fewer wavelengths. Thus one can conclude that it is very necessary to first perform wavelength selection before building a calibration model. The two methods had achieved almost the same good prediction results (SECV and R_P,CV_). However, the optimal OPWC-PLS model adopted only 28 wavelengths, while the other adopted 159 wavelengths. Therefore, the OPWC method has great prediction performance for wavelength selection.

The differences in prediction of the OPWC-PLS and MW-PLS methods for GLB illustrate that MW-PLS can achieve higher prediction accuracy, but it is time-consuming and employs more wavelengths, while OPWC-PLS can achieve similar prediction results with MW-PLS in less time. In addition, MW-PLS, as a continuous wavelength screening method, is more suitable for determining the object with relatively concentrated molecular absorption bands; while OPWC-PLS, as a discrete wavelength screening method, may be more suitable for determining the object with relatively fragmented molecular absorption bands.

## Conclusion

The change of GLB content in human serum has important reference value for clinical trial and disease diagnosis. In this study, the OPWC-PLS method was employed for rapid analysis of GLB based on NIR spectroscopy. MW-PLS and MC-UVE-PLS methods were also employed for comparison. The results indicate that, OPWC-PLS and MW-PLS methods achieved satisfactory prediction results, while the MC-UVE-PLS method was not suitable for the data set of this study, and the prediction effect of the model is not significantly improved. The optimal OPWC-PLS model adopted 28 wavelengths, and corresponding SECV and R_P,CV_ were 0.813 g L^−1^ and 0.978, respectively. The optimal MW-PLS model adopted 159 wavelengths, and corresponding SECV and R_P,CV_ were 0.804 g L^−1^ and 0.979, respectively. The OPWC-PLS achieved almost the same prediction effect as MW-PLS with faster speed and fewer wavelengths. Therefore, OPWC is an efficient approach for information wavelength selection.

The predicted GLB values obtained by MW-PLS and OPWC-PLS were highly correlated with the reference values. Compared with traditional method, the method based on NIR spectroscopy has the merits of rapidity, simplicity and no chemical reagent. Therefore, the results have important reference value for the rapid determination of GLB. In addition, the wavelengths selected by the two methods are partially the same, reflecting the commonness and difference of different methods.

## Data Availability

The datasets used and/or analysed during the current study are available from the corresponding author on reasonable request.

## Abbreviations

GLB: Globulin
NIR: Near infrared
OPWC: Optimal partner wavelength combination
MW-PLS: Moving window partial least squares
MC-UVE: Monte Carlo uninformative variable elimination
SECV: Root-mean-square error of cross validation of prediction
R_P,CV_: Correlation coefficient of prediction
BLR: Binary linear regression
PWS: Partner wavelength subset
LOOCV: Leave-one-out cross validation
SD: Standard deviation
